# Evaluating physical urban features in several mental illnesses using electronic health record data

**DOI:** 10.3389/fdgth.2022.874237

**Published:** 2022-09-07

**Authors:** Zahra Mahabadi, Maryam Mahabadi, Sumithra Velupillai, Angus Roberts, Philip McGuire, Zina Ibrahim, Rashmi Patel

**Affiliations:** ^1^ Centre for Urban Science and Progress, King’s College London, London, United Kingdom; ^2^ Warwick Manufacturing Group, University of Warwick, Coventry, United Kingdom; ^3^ Department of Psychosis Studies, Institute of Psychiatry, Psychology, and Neuroscience, King’s College London, London, United Kingdom; ^4^ Health Data Research UK, London, United Kingdom; ^5^ NIHR Maudsley Biomedical Research Centre, South London and Maudsley NHS Foundation Trust, London, United Kingdom; ^6^ Department of Biostatistics & Health Informatics, King’s College London, London, United Kingdom

**Keywords:** geospatial informatics, schizophrenia, bipolar disorder, psychosis, machine learning, ehr (electric heath record)

## Abstract

**Objectives:**

Understanding the potential impact of physical characteristics of the urban environment on clinical outcomes on several mental illnesses.

**Materials and Methods:**

Physical features of the urban environment were examined as predictors for affective and non-affective several mental illnesses (SMI), the number and length of psychiatric hospital admissions, and the number of short and long-acting injectable antipsychotic prescriptions. In addition, the urban features with the greatest weight in the predicted model were determined. The data included 28 urban features and 6 clinical variables obtained from 30,210 people with SMI receiving care from the South London and Maudsley NHS Foundation Trust (SLaM) using the Clinical Record Interactive Search (CRIS) tool. Five machine learning regression models were evaluated for the highest prediction accuracy followed by the Self-Organising Map (SOM) to represent the results visually.

**Results:**

The prevalence of SMI, number and duration of psychiatric hospital admission, and antipsychotic prescribing were greater in urban areas. However, machine learning analysis was unable to accurately predict clinical outcomes using urban environmental data.

**Discussion:**

The urban environment is associated with an increased prevalence of SMI. However, urban features alone cannot explain the variation observed in psychotic disorder prevalence or clinical outcomes measured through psychiatric hospitalisation or exposure to antipsychotic treatments.

**Conclusion:**

Urban areas are associated with a greater prevalence of SMI but clinical outcomes are likely to depend on a combination of urban and individual patient-level factors. Future mental healthcare service planning should focus on providing appropriate resources to people with SMI in urban environments.

## Background

Several metal illnesses (SMI) (1) such as schizophrenia, schizoaffective disorder, bipolar disorder, and psychotic depression are serious mental illnesses that have a substantial impact on all aspects of daily life (2). Schizophrenia and bipolar disorder were ranked as the top 15 and 23 leading causes of global disability, respectively, in 2016 (3). While they are classified as low prevalence disorders (4), their impact on functioning and quality of life leads to considerable social and economic costs at local, national, and international levels. More specifically, the economic burden attributed to SMI is estimated to range from 0.02% to 1.65% of GDP (Gross Domestic Product) across the globe (5).

There is a need to better understand risk factors associated with SMI to develop effective strategies for the prevention of poor clinical outcomes and relapse. Although the precise causes and the aetiology of SMI are not well characterised (6), several studies have implicated risk factors such as gender, age, substance abuse, prenatal exposure to infections, neurodevelopmental disorders, and maternal stress (6–8). In general, previous studies have classified risk factors into two main groups: genetic and environmental factors. While many heritability studies support a major role for genes to contribute up to 80% of the liability for developing psychosis (7), Susser and Schwarts (9) claimed that these studies underestimate the role of the environment. Brown (10) also enumerated several reasons, such as the difficulty in measuring the gene-environment interaction, which heritability models contribute to an underestimation of the contribution of environmental risk factors.

## Significance: environment features and psychosis relapse

More recently, the role of the environment in vulnerability to psychosis has received increasing attention. Urban birth, upbringing, and migration to the urban area can increase the likelihood of psychosis (7). Considering that genetic factors may not be the sole determinant of onset and clinical outcomes in people with SMI, studying urban features could help to better understand the associated environmental risk factors.

In this study, electronic health record (EHR) data of people with schizophrenia, schizoaffective disorder, and bipolar disorder, which are the most common SMI (11), were investigated according to the physical features of urban areas. Studying urban features related to psychosis can be classified into two groups: between-cities (urbanization level) and within-cities (neighbourhood level) (12–14). Studies related to the first groups show that an increased risk of psychosis is associated with being raised or living in urban areas, compared to rural areas (12, 15–17). Based on the results of a large study conducted in Denmark, the risk of schizophrenia is two-fold greater among individuals who were born in the capital city compared to rural regions (18). The second group of urban-oriented studies includes studies that examine neighbourhood characteristics by considering socioeconomic features such as ethnic fragmentation, social capital, deprivation, social isolation, and poverty. Many studies highlight the contribution of socioeconomic features to the elevated risk of psychosis across different neighbourhoods (13, 15, 19), while one of them was focused on immigrants (20). In a study examining new cases over a 7-year interval in the Netherlands, a significantly increased incidence of SMI was reported among ethnic minorities in low-ethnic-density neighbourhoods in comparison to high-ethnic-density neighbourhoods (21).

All of these studies reported a higher prevalence of psychotic disorders in urban areas in comparison to suburban and rural areas, while they investigated the impact of the urban features from demographic, social, and economic viewpoints. However, urban areas are also distinguishable by considering their physical features. For instance, central areas in cities are usually made up of higher and bigger buildings, a smaller proportion of green spaces per capita, denser amenities, and higher road traffic (22).

## Objectives

We sought to conduct an in-depth investigation into the impact of physical features of an urban environment on the relapse of SMI by linking clinical data from a de-identified EHR dataset with London geospatial datasets. We selected six clinical variables from a large-scale EHR dataset and 28 urban features from geospatial datasets using machine learning models to examine the potential association between urban and clinical data. In this regard, we sought to address the following questions; (i) how are physical urban features associated with the prevalence of SMI? (ii) are physical urban features associated with clinical outcomes measured through the number and duration of psychiatric hospital admissions? (iii) are physical urban features associated with prescribing short and long-acting antipsychotics? The 28 physical urban features were selected based on the different urbanisation levels established by the transect planning approach (23).

## Materials and methods

We trained five regression models to investigate associations between clinical labels and physical urban features, visualising the investigation using Self-Organising Maps (SOM) to demonstrate the association or disassociation between these two sets of data. We repeated the experiments using five regression models to ensure that the results are model agnostic and are generalizable. Urban data were obtained at Lower Layer Super Output Area (LSOA) level. The LSOA refers to a discrete geographical area representing around 650 households (24). The study area included four boroughs in the southern part of London; Croydon, Lambeth, Southwark, and Lewisham which cover 733 LSOAs. All five models were trained using urban data as features and clinical variables as outcome labels. In the following sections, we describe how clinical and urban data were prepared and analysed in the regression models.

### Clinical data

Clinical data were extracted from the South London and Maudsley (SLaM) NHS Foundation Trust Clinical Record Interactive Search tool (CRIS) which comprises de-identified, structured, and free-text data derived from the EHR dataset used by SLaM, a large mental healthcare provider in southeast London (25). The most recent update of the data used in this study was in June 2019. The dataset included 260 clinical labels for 30,210 patients with schizophrenia, schizoaffective disorder, or bipolar disorder. Approval for the project was obtained through a local Oversight committee that governs access to the CRIS dataset for research as a de-identified electronic case register for secondary analysis. Ethical approval for the CRIS dataset has been obtained from the Oxford C Research Ethics Committee (18/SC/0372).

#### Clinical labels

Clinical labels were selected and extracted from the CRIS data for further analysis *via* the following procedures:

a. Prevalence of SMI per LSOA: The prevalence of psychosis was estimated by counting the number of patients in each LSOA according to the two groups of affective (schizoaffective disorder and bipolar disorder) (26) and non-affective psychosis (schizophrenia).
b. The number and duration of psychiatric hospitalisation: The mean number of psychiatric hospital admissions and days spent in the hospital under a Mental Health Act section were estimated. The follow-up duration varied with different patients; some of them had long and some of them had shorter follow-up durations. Therefore, the number of hospital admissions and days spent in the hospital were divided by the number of months from the earliest recorded diagnosis to June 2019 and then aggregated for each LSOA.
c. The number of short and long-acting antipsychotic prescriptions: Antipsychotic prescriptions were calculated according to the two classes of antipsychotics, and then aggregated for each LSOA;
  • Short-acting (non-LAI) = 1st generation short-acting Antipsychotic + 2nd generation short-acting Antipsychotic
  • Long-acting Injectable (LAI) = 1st generation long-acting Antipsychotic + 2nd generation long-acting Antipsychotic

Since different LSOAs had different populations, all labels were standardised by dividing by the population of the respective LSOA. The final output is the following 6 clinical labels;

1. Exposure to non-LAI antipsychotic prescriptions
2. Exposure to LAI antipsychotic prescriptions
3. Mean number of days spent in hospital since the earliest recorded diagnosis per month
4. Mean number of hospital admissions since the earliest recorded diagnosis per month
5. Prevalence of non-affective psychosis
6. Prevalence of affective psychosis

### Physical urban data

**Figure 1:**
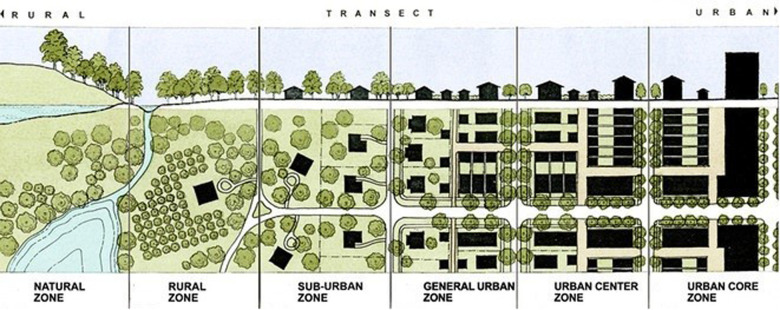
Transect planning approach (22).

Physical urban data were prepared within a geographical cross-section from the central areas of London to the suburbs to examine the sequence of different physical characteristics which comprise the built environment according to the transect planning approach (23). This approach represents the transition from central, urbanised areas to rural and natural zones outside the suburbs (Figure 1). (22) Urban features characterised within this approach include buildings, open spaces, roads, and types of land use (27).

Urban features were prepared for analysis using Python 3.0 and QGIS 3.0 to collate data on the following attributes:

1. Height of buildings: The data of the buildings’ height were obtained from the Ordnance Survey (28), through the Digimap website (23). The most recent update was October 2017 (24). Buildings in central areas of cities are taller than buildings in suburbs.
2. Footprint area of buildings: This feature evaluates the degree to which geospatial areas are occupied by buildings. Central areas typically have smaller open spaces due to a greater proportion of space being occupied by buildings. Footprint area of buildings data was obtained from the Ordnance Survey (28) height of buildings dataset (24).
3. The number of points of interest (POI): POIs consist of all public and privately owned businesses, education, and leisure services (28). The density of POIs is usually greater in central areas in comparison with suburban areas. The Ordnance Survey POIs dataset was used, accessed from the Digimap website and the most recent update was in September 2018 (24). The following nine POI classes were extracted from the dataset, presented in Table 1, below.
4. Space Syntax Index: The degree of centrality that each road has within a road network can be represented through a Space Syntax Index (29). The Space Syntax data were provided by Space Syntax Limited, accessible from Space Syntax Open Mapping (30). The most recent update was in March 2018 (31). For this project, the 10 km scale was chosen among three available scales of 2, 10, and 100 km, to emphasise vehicle movement which has a greater role in defining road usage in urbanized areas. Two of the main indices of space syntax which were used in this project are as the following (32).
d. Accessibility: This is based on the network measure of Closeness Centrality. The higher the score, the more accessible the road is. Since roads in central areas are denser and linked to other roads, by having more intersections, these roads are more accessible than roads in the suburbs (31).
e. Connectivity: This is based on the network measure of Between-ness Centrality which measures how often a road lies within the shortest path between any pair of other roads. Since roads in suburbs usually play a role as highways, they have higher connectivity scores (32).
5. Green space area: Because of the compact morphology of central areas with the dominant role of the built environment, the proportion of green spaces per capita is generally smaller in central areas (23). Greenspace data were obtained from the Ordnance Survey accessed from the Digimap website and the most recent update was in April 2019 (24)
6. Public Transport Access Level (PTAL): This measure rates a selected place based on how close it is to public transport and how frequently services are in the area (33). The number and frequency of public transport are usually higher in central areas, to provide services for more populated areas (33). The PTAL dataset, provided by Transport for London (TfL), was accessed from the London Datastore website (34). The current base year to calculate PTAL scores is the 2011 census (33).
7. Dwelling type: This includes a breakdown by detached, semi-detached, terraced, and flat/maisonette (35). Across the urban-rural transect spectrum, the dominance of dwelling types changes from detached and semi-detached in suburbs to terraced and flat/maisonette in central areas (34). The Dwelling type dataset, provided by Valuation Office Agency, was accessed from the London Datastore website (36) and the most recent update was in March 2015.
8. Dwelling age: Buildings located relatively far from central areas are generally older than buildings located in central areas, due to the construction boom in these areas. However, this trend was not observed in the study geographic area, potentially due to planning and building restrictions in historic areas of central London. The Dwelling age dataset, provided by Valuation Office Agency, was accessed from the London Datastore website (36) and the most recent update was in March 2015. The original dataset contained 12 periods with approximately 10-year intervals, from pre-1900 to 2014. This project was split into three periods; from pre-1900 to 1939, from 1945 to 1999, and from 2000 to 2014.

**Table 1:** POIs classifications.

Digit code	POI class
01	Accommodation, Eating and Drinking
02	Commercial Services
03	Attractions
04	Sport and Entertainment
05	Education and Health
06	Public Infrastructure
07	Manufacturing and Production
09	Retail
10	Transport

The height, footprint area of the buildings, and Space Syntax datasets have a large variance. For example, there were very tall buildings as well as very short buildings in certain LSOAs. Thus, calculating the mean or median height of the buildings for each LSOA could result in losing much information. For this reason, these three urban features were classified into 5 classes from very short/small/low to very tall/large/high, respectively. This classification was by using natural break classification in QGIS based on the Jenks Natural Breaks algorithm. Class breaks are identified such that the best groups have similar values and maximize the differences between classes (37).

Since different LSOAs had different areas, the above-mentioned features were standardised. Space Syntax indices were divided by the length of roads in each LSOA while the other feature was divided by the area of the respective LSOA. The final 28 features included in the study are presented in Table 2.

**Table 2:** Classification of urban features.

**Urban features**	** **	**28 final classes**
Height of buildings		1. Mean number of very short buildings in one squared meter of each LSOA 2. Mean number of short buildings in one squared meter of each LSOA 3. Mean number of medium-height buildings in one squared meter of each LSOA 4. Mean number of tall and very tall buildings in one squared meter of each LSOA
Footprint area of buildings		5. Mean area of very small buildings in one squared meter of each LSOA 6. Mean area of small buildings in one squared meter of each LSOA 7. Mean area of medium size buildings in one squared meter of each LSOA 8. Mean area of large and very large buildings in one squared meter of each LSOA
The number of POIs		9. Mean number of Accommodation, Eating, Drinking, Attractions, and Retail in one squared meter 10. Mean number of Commercial Services, Sport and Entertainment, and Public Infrastructure in one squared meter 11. Mean number of Education and Health in one squared meter
Space Syntax Indexes	Connectivity	12. Mean length of roads with very low spatial connectivity in each LSOA 13. Mean length of roads with low spatial connectivity in each LSOA 14. Mean length of roads with medium spatial connectivity in each LSOA 15. Mean length of roads with high and very high spatial connectivity in each LSOA
	Accessibility	16. Mean length of roads with very low spatial accessibility in each LSOA 17. Mean length of roads with low spatial accessibility in each LSOA 18. Mean length of roads with medium spatial accessibility in each LSOA 19. Mean length of roads with high spatial accessibility in each LSOA 20. Mean length of roads with very high spatial accessibility in each LSOA
Green space area		21. Mean area of green space in one squared meter of each LSOA
Public Transport Access Level (PTAL)		22. Mean public transport access level in each LSOA
Dwelling type		23. Mean number of dwellings with the type of flat/maisonette in one squared meter of each LSOA 24. Mean number of dwellings with the type of terraced in one squared meter of each LSOA 25. Mean number of dwellings with the type of detached and semi-detached in one squared meter of each LSOA
Dwelling age		26. Mean number of dwellings built pre 1900 and 1939 in one squared meter of each LSOA 27. Mean number of dwellings built between 1945 and 1999 in one squared meter of each LSOA 28. Mean number of dwellings built between 2000 and 2014 in one squared meter of each LSOA

### Regression models for numerical exploration

Before developing regression models to investigate associations between clinical labels and physical urban features, the input variables were pre-processed to normalise the data, detect outliers in clinical variables, and remove collinear features. All features and labels were normalized by the following formula (where x represents the raw feature/label data and z represents the normalised feature/label data) (38).


$$z=\frac{x-\min\left(x\right)}{\max\left(x\right)-\min\left(x\right)}$$

Outliers were detected by estimating the double median absolute deviation (39). This method is suitable for asymmetrical distributions (40) as the clinical labels had skewed distributions. To find multi-collinearity, Pearson's correlation was applied where the threshold of 70% was defined to find very collinear features. As a result, the mean number of POIs (Commercial Services. Sport and Entertainment, Public Infrastructure) and dwelling type (Flat_Mais) were removed, leaving 26 features to be applied in the following steps; 1- feature selection, 2- performance metrics, and 3- prediction models.

#### Feature selection

We performed feature selection using LASSO (Absolute Shrinkage and Selection Operator), which is a frequently used method having known good prediction accuracy (41). It is based on the idea that the most influential features have the highest LASSO coefficients, while the uncorrelated features have coefficients close to zero (42). We Least Absolute Shrinkage and Selection Operator Cross-Validation (LASSOCV) available in Python's sklearn package, which implements a Lasso linear model with iterative fitting along a regularization path. We implemented a 10-fold cross validation, using 10 different tolerance thresholds for the objective function ranging from 0.0001 to 1.

#### Performance metrics

Different performance metrics can be used to compare the results of prediction models with the actual (observed) data. In this study, two primary performance metrics (43) were used: Mean Absolute Error (MAE) and Accuracy. MAE is based on the absolute value of the difference between the predicted value and the actual value (43). The Accuracy was estimated as Accuracy = 100—Symmetric mean absolute percentage error (SMAPE) where SMAPE was estimated using the equation below (44). Both are scale dependent with linear error scale and capable of dealing with zero values in the actual data:

$$\begin{array}{rl} \mathrm{SMAPE} & =100\;\mathrm{MEAN}\\ & \;(2*\;\frac{\left|\mathrm{actual}\;\;\mathrm{values}-\;\mathrm{predicted}\;\mathrm{values}\right|}{\left|\mathrm{actual}\;\mathrm{valactual}\;\mathrm{values}+\;\mathrm{predicted}\;\;\mathrm{valuesues}\right|} \end{array}$$

#### Prediction models

Each of the regression tasks was implemented using five different algorithms (45) *via* the Python 3.0 package, Scikit-learn. 1- Multiple Linear regression, 2- Random forest regression, 3- Gradient boosting regression, 4- Support vector regression, and 5- K-nearest neighbour's regression.

Feature importance refers to how important is the role of a feature in determining the output of the prediction models. A feature is “important” if changing its values induces a significant change in the model error (46). We examined feature importance with respect to each model's output by comparing the statistical correlation scores assigned to input features in the models producing the best results

### Examining complete spatial randomness (CSR)

**Figure 2:**
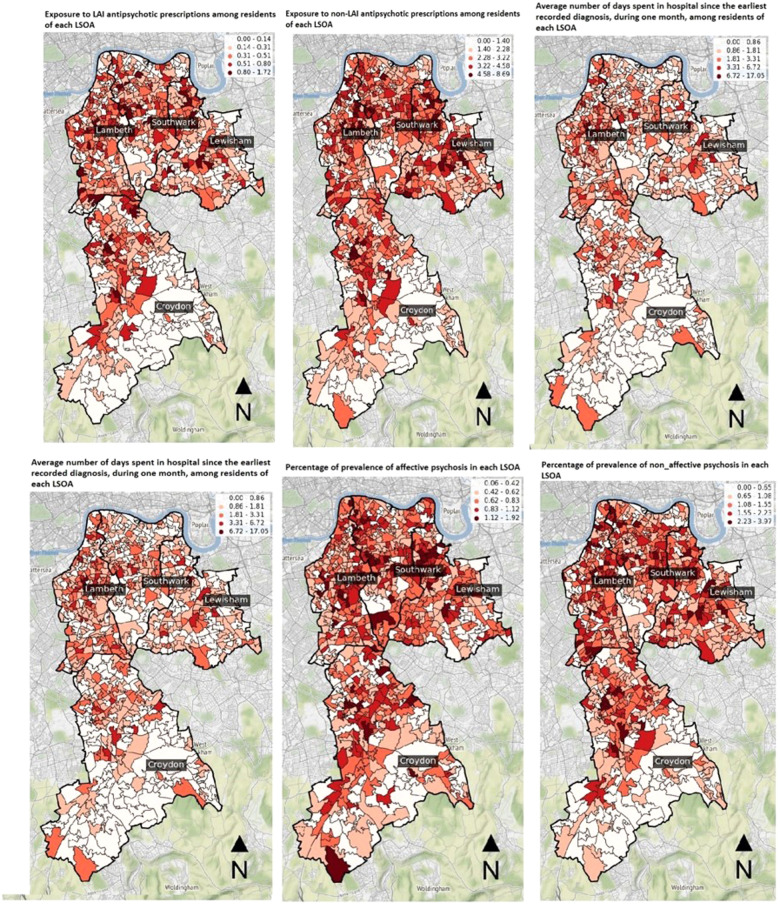
Visualising the distribution of clinical labels.

We examined if the distribution of clinical data in the study area was either random or followed a specific pattern. If it were random, it would indicate that there is no spatial interaction between the clinical data and their geographic locations (47). We visually inspected the clinical data on maps (refer to Figure 2) and applied CSR to evaluate whether or not an observed pattern of distribution within a spatial area occurred at random using the quadrat analysis test. If the p-value of the quadrat analysis test is less than 0.00001, the null hypothesis of spatial randomness can be rejected showing the point pattern is not random (48).

### SOM for graphical exploration

SOM is an unsupervised neural network method transforming high-dimensional data to a low-dimensional grid of nodes to map similar samples to each node and represent their distribution (49). SOM was applied to demonstrate the potential association between physical and clinical data, visually. Similar LSOAs were assigned to each node in a 7*7 grid, based on their closeness and similarity of the urban physical features with minimum objects distance and neighbours' distance. Objects distance refers to the mean distance of objects mapped to a unit to the codebook vector of the respective unit and neighbours' distance shows the sum of the distances to all immediate neighbours (50). Clinical variables were assigned to their respective nodes according to their LSOA codes to compare their distributions with urban physical features. To implement SOM, the Kohonen package in R 3.6.0 was applied.

## Results

Six maps in Figure 2 show the non-random distribution of clinical data in the study area. As depicted, the number of LSOAs with darker colours is higher in the northern part of the study area which is closer to central London while lighter colour LSOAs are higher in the southern area. Thus, geospatial visualisation of clinical data indicated that the prevalence of affective and non-affective psychosis was higher in more central, Northern LSOAs compared to suburban, Southern LSOAs. Likewise, the number of patients exposed to LAI and non-LAI antipsychotic prescriptions, as well as the number and duration of psychiatric hospitalisation, were significantly different between Northern and Southern LSOAs. These differences were corroborated by CSR analysis which revealed a non-random special distribution of all selected clinical labels (Table 3). Since all p-values are smaller than 0.05, the null hypothesis of CSR can be rejected showing there could be any association between the selected physical features of urban environment and the non-random distribution of clinical labels.

**Table 3:** CSR examination.

**CSR test**	**The chi-squared test statistic for the observed point pattern**	**P-value for Chi-squared test statistic**
CSR test for exposure to non-LAI antipsychotic prescriptions	13,920.14	<0.000001
CSR test for exposure to LAI antipsychotic prescriptions	1,647.51	<0.000001
CSR test for average number of days spent in hospital	8,956.21	<0.000001
CSR test for average number of hospital admissions	196,372.50	<0.000001
CSR test for percentage of prevalence of non-affective psychosis	114,925.25	<0.000001
CSR test for percentage of prevalence of affective psychosis	59,468.90	<0.000001

Table 4 shows the results of conducting the feature importance process. Six of the most influential urban features were identified as they had the greatest magnitude of correlation scores in the prediction models. Four of these urban features had a negative correlation with clinical labels while two of the urban features had a positive correlation.

**Table 4:** Comparing coefficients.

*Clinical labels*	*Urban features*	*Coefficients*
*Exposure to non-LAI Antipsychotic Prescriptions*	Mean number of Dwellings built between 2000 and 2015	−0.54
	Mean number of detached-type dwellings	−0.26
	Mean number of tall and very tall buildings	−0.19
	Mean area of green space	−0.18
	Mean area of very small buildings	+0.15
	Mean public transport access level	+0.19
*Prevalence of non-Affective Psychosis*	Mean number of Dwellings built between 2000 and 2015	−0.66
	Mean number of detached-type dwellings	−0.34
	Mean number of tall and very tall buildings	−0.18
	Mean area of green space	−0.17
	Mean area of very small buildings	+0.14
	Mean public transport access level	+0.20
*Prevalence of Affective Psychosis*	Mean number of Dwellings built between 2000 and 2015	−0.43
	Mean number of detached-type dwellings	−0.24
	Mean number of tall and very tall buildings	−0.18
	Mean area of green space	−0.17
	Mean area of very small buildings	+0.13
	Mean public transport access level	+0.31

**Figure 3:**
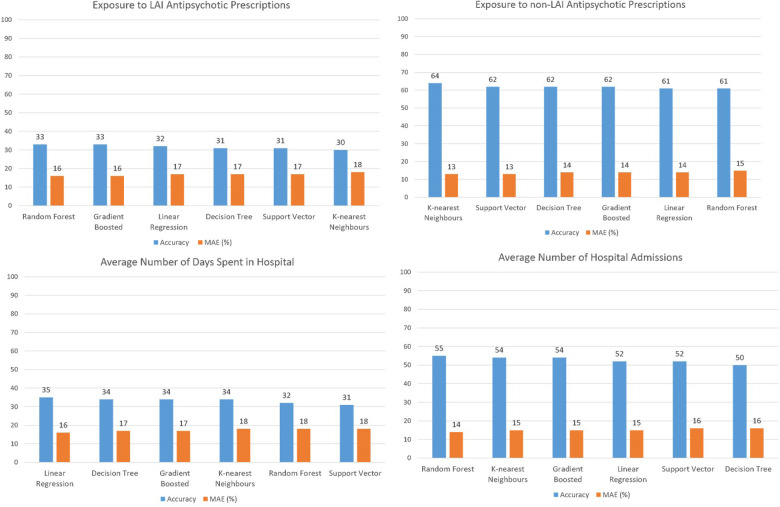
Models output comparison.

**Figure 4:**
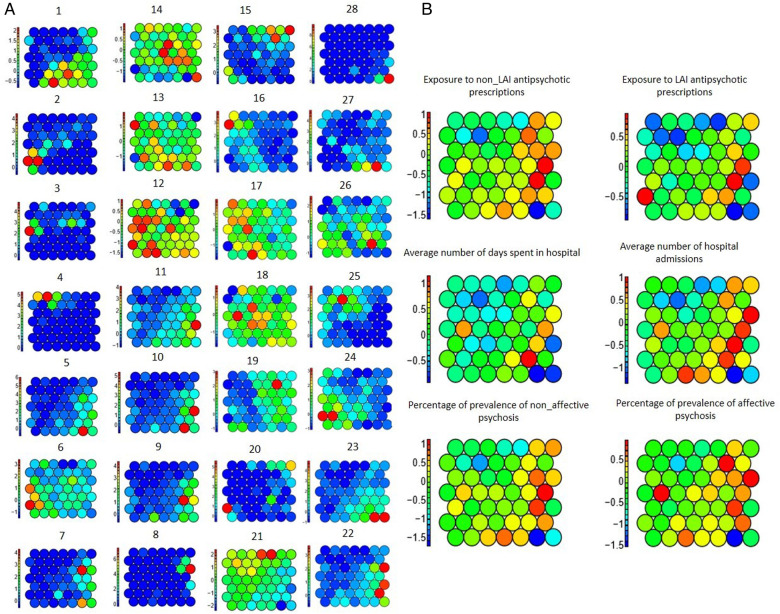
SOM of physical features on the left-hand side (**A**) and clinical labels on the right-hand side (**B**); 1–28 are based on the numbers assigned to physical features in table 2.

Five regression models were applied to examine whether the distribution of clinical labels was associated with the distribution of physical characteristics of the urban environment across a cross-section from the central areas of London to the suburbs. Figure 3 illustrates the results of the regression models. The linear regression model produced the best result for three clinical labels. Random forest produced the highest accuracy and lowest error for two clinical labels and k-nearest neighbours made the best results for one clinical label. However, there was no significant difference between the results of the five regression models. The prediction of three clinical labels including (i) prevalence of Affective Psychosis, (ii) exposure to non-LAI antipsychotic prescriptions, and (iii) prevalence of non-Affective Psychosis showed the highest accuracy and lowest error, respectively. The graphical outputs of SOM (Figure 4) also confirmed the numerical results of regression models as there were not similar colour patterns between the distributions of 28 urban physical features (on the left hand Sid of Figure 4) and 6 clinical labels (on the right hand Sid of Figure 4).

## Discussion

### Main findings

The present study conducted an in-depth investigation to evaluate the prediction of SMI and their derivatives based on the physical features of the urban environment. We found that LSOA-adjusted prevalence of affective and non-affective SMI, prescription of antipsychotics, and psychiatric hospitalisation were greatest in urban areas compared to suburban areas. This is in keeping with previous studies which have demonstrated an increased risk of psychosis associated with living in urban areas (12, 15–17).

However, no machine learning models were able to accurately predict these clinical features based on urban data as defined by physical features corroborated by SOM. The highest prediction accuracy was around 70%. Similarly, the greatest coefficient for the most important urban feature was 0.66 which is too low to be considered as an influential feature on prediction outcome. For this reason, we were unable to use the physical features of the urban environment to predict clinical outcomes of SMI. This could be because the most important determinants of clinical outcomes in people with SMI relate to individual patient-level factors such as genetic factors, treatment adherence, illicit substance use and stressful life events which were not captured in the clinical data analysed in this study (6–9).

### Strengths and limitations

We analysed a large repository of clinical data derived from electronic health records, representative of real-world clinical practice, together with geospatial data derived from publicly available datasets characterising a range of physical urban features relevant to risk factors that may contribute towards psychotic disorder onset and clinical outcomes. To the best of our knowledge, this is the first study to combine real-world data from healthcare services and national geospatial datasets in a sample size of over 30,000 patients with SMI. We developed novel methods to analyse clinical and physical urban feature data at the LSOA level. These methods could be applied to investigate the relationship between physical urban features and clinical outcomes in other mental disorders.

The use of LSOA to define geospatial areas may have limited the ability of machine learning analyses to accurately predict clinical labels due to the resolution of scale to define geographic location. As LSOA level data includes several hundred households, physical geospatial data may vary within each LSOA leading to heterogeneity in exposure to urban risk factors. This could be particularly problematic in central areas of London where this study was conducted where a single LSOA may encompass wide ranging sociodemographic factors and urban features. Conducting the same analysis at higher geospatial resolution (such as postcode) may reveal more meaningful associations between urban features and clinical outcomes.

### Implications

The urban environment is associated with an increased prevalence of SMI. However, urban features alone cannot explain the variation observed in psychotic disorder prevalence or clinical outcomes measured through psychiatric hospitalisation or exposure to antipsychotic treatments. Future studies which include additional patient-level factors such as treatment adherence, illness severity and exposure to illicit drugs and greater special resolution of urban feature data could enable a more accurate prediction of clinical outcomes in people with psychotic disorders.

## Data Availability

The original contributions presented in the study are included in the article/Supplementary Material, further inquiries can be directed to the corresponding author/s.
